# Flower electrodes for comfortable dry electroencephalography

**DOI:** 10.1038/s41598-023-42732-8

**Published:** 2023-10-03

**Authors:** Indhika Fauzhan Warsito, Milana Komosar, Maria Anne Bernhard, Patrique Fiedler, Jens Haueisen

**Affiliations:** 1 Institute of Biomedical Engineering and Informatics at the Technische Universität Ilmenau, Ilmenau, Germany; 2 Department of Neurology, Biomagnetic Center, University Hospital Jena, Jena, Germany

**Keywords:** Brain imaging, Biomedical engineering

## Abstract

Dry electroencephalography (EEG) electrodes provide rapid, gel-free, and easy EEG preparation, but with limited wearing comfort. We propose a novel dry electrode comprising multiple tilted pins in a flower-like arrangement. The novel Flower electrode increases wearing comfort and contact area while maintaining ease of use. In a study with 20 volunteers, we compare the performance of a novel 64-channel dry Flower electrode cap to a commercial dry Multipin electrode cap in sitting and supine positions. The wearing comfort of the Flower cap was rated as significantly improved both in sitting and supine positions. The channel reliability and average impedances of both electrode systems were comparable. Averaged VEP components showed no considerable differences in global field power amplitude and latency, as well as in signal-to-noise ratio and topography. No considerable differences were found in the power spectral density of the resting state EEGs between 1 and 40 Hz. Overall, our findings provide evidence for equivalent channel reliability and signal characteristics of the compared cap systems in the sitting and supine positions. The reliability, signal quality, and significantly improved wearing comfort of the Flower electrode allow new fields of applications for dry EEG in long-term monitoring, sensitive populations, and recording in supine position.

## Introduction

Dry electrodes for electroencephalography (EEG) have contributed to significantly reducing preparation and cleaning effort for EEG acquisitions in comparison to conventional gel-based electrodes. Moreover, dry electrodes can be self-applied without the need for trained medical staff and the risk of falsified measurements due to incorrect gel application. State-of-the-art dry EEG electrodes enable fast application^1–5^ and support new fields of application such as mobile EEG^2,6^, emergency and prehospital care^7–9^, brain-computer interfaces^10–13^, neurofeedback^14–17^, and clinical trials^18^.

The primary requirements for the shape of dry electrodes include (i) allowing passing through the hair layer, (ii) establishing reliable contact with the scalp, (iii) adapting to individual head curvature, and (iv) distributing pressure evenly and avoiding spots of excessive local pressure. Several electrode models have been studied previously, comprising either multiple straight pins^2,4,5,19–25^, bristles^11^, combinations of pins and bristles^26^, spider-like pin arrangements^27–29^, twisted pins^29^, or arch shapes^30,31^. Commercially available electrodes, such as waveguard touch (ANT B.V., Hengelo, The Netherlands), Drytrode (Neuroelectrics Barcelona SLU, Barcelona, Spain), SoftPulse (Dätwyler Inc., Altdorf, Switzerland), g.SAHARA and g.SAHARA Hybrid/Unicorn Hybrid Black (g.tec medical engineering GmbH, Schiedlberg, Austria) implement designs with multiple straight pins. The CGX Flex sensor (Cognionics, California, USA) implements a spider-like pin arrangement.

The requirement to have an optimal electrode-scalp contact usually conflicts with the wearing comfort or easy (self-)application. As a result, low comfort is a significant issue for state-of-the-art dry EEG electrodes^2,4,5,18,25,31–33^ and therefore limits or impedes their use on sensitive populations like neonates or infants, application in supine position, and long-term recordings. Improvements in the wearing comfort of dry electrodes are desirable, especially for non-clinical EEG applications, like brain-computer interfaces, neurofeedback, and in home applications^34–36^. Moreover, comfortable recordings in supine positions would contribute to simplified use in medical diagnostics e.g., in sleep studies^37–41^ neonatal care^42–45^, and multimodal recordings combining EEG with e.g., magnetic resonance imaging (MRI) or magnetoencephalography (MEG). Until now, EEG studies with dry electrodes have been conducted in supine position only for low-density setups such as dry ear-EEG^39,41^, flat electrodes on the hairless forehead^37,38,40^, and headbands with the dry electrodes^46^.

Multipin electrodes have been successfully validated previously for multi-channel EEG in single-center^1,2,4,25^, and multi-center studies^5^. We also investigated the optimal parameters of pin number, flexibility, and adduction pressure to balance wearing comfort and impedance in single^47^ and multi-channel setups^25^. Moreover, it was shown that an increase in wearing comfort can be achieved in multi-channel setups by using spring-loaded or sponge-based mechanisms^24,32^. However, the increased comfort did not reach the level required for repetitive long-term use^5,25^ and recordings in supine position. Often, increased comfort was achieved while compromising ease or speed of application^24,31^.

We propose a novel dry electrode design—which we call the Flower electrode. The development followed a bionic approach with the aim of considerably improving wearing comfort during recordings in sitting and supine positions, while maintaining the speed of application, channel reliability and signal quality. The Flower electrode consists of 30 tilted pins on one common baseplate, arranged in a Daisy-flower-like intertwined arrangement. The electrode substrate is implemented using vacuum casting by means of a flexible, biocompatible polymer with adjustable hardness. Due to the pin shape, size, angle, and arrangement, the Flower electrode design increases scalp contact area and shows increased conformability to the individual head shape.

In this study, we describe the design of the novel Flower electrode and investigate its performance in comparison to commercial dry Multipin electrodes with straight pins within 64-channel textile caps on 20 healthy volunteers of different sex and hair length. We examine performance metrics including preparation time, wearing comfort, electrode–skin impedance, channel reliability, and EEG signal quality for the standard EEG bands between 1 and 40 Hz. EEG with both caps was recorded sequentially for each volunteer. The EEG paradigm is based on previous comparative dry EEG studies^2,4,5,25,31,48^ in sitting position, adding extra performance assessment in supine position.

## Materials and methods

### EEG electrodes and caps

**Figure 1:**
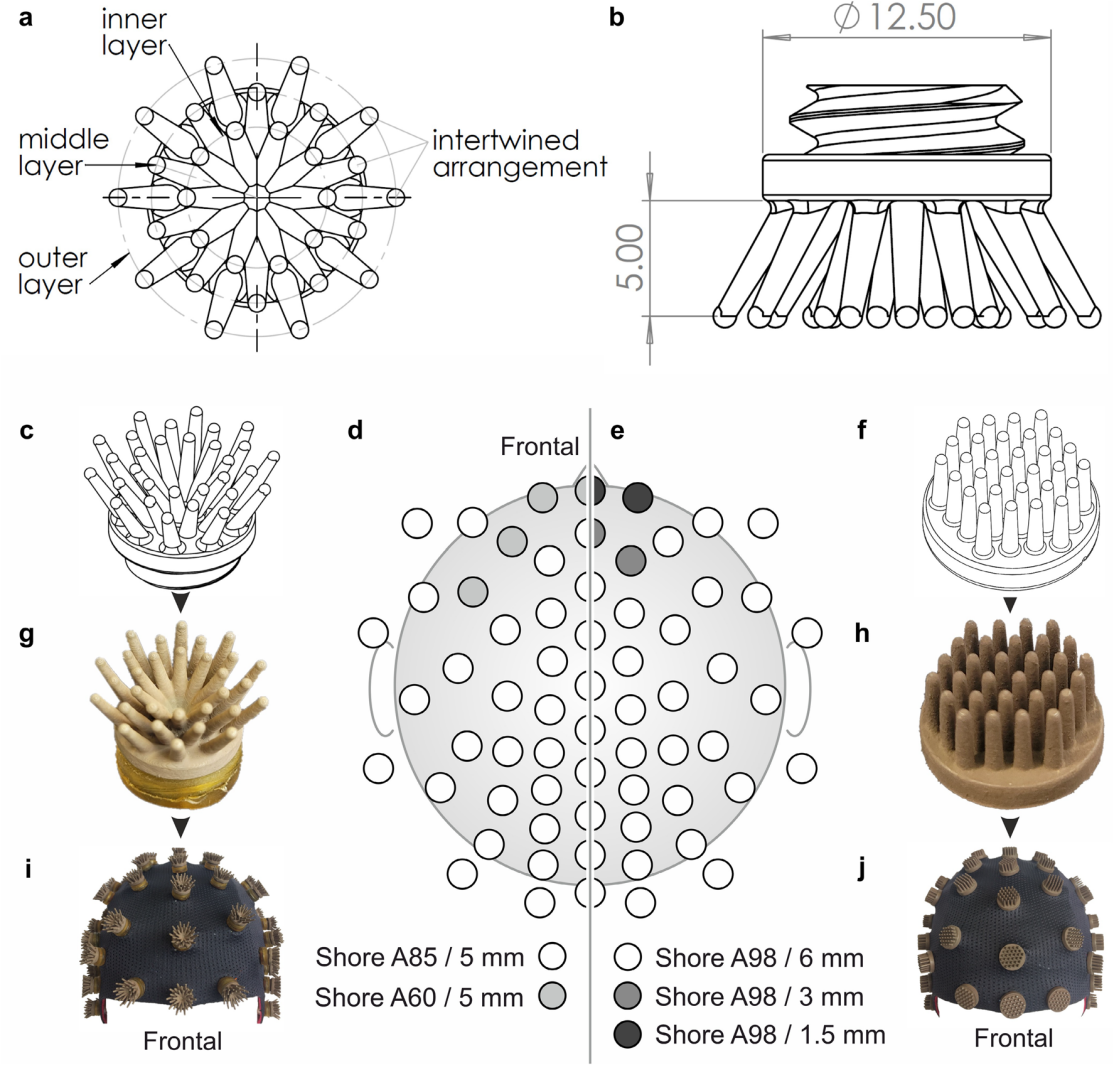
64 channel dry electrode caps: Flower electrode schematic shown in (**a**) bottom and (**b**) side view. The angled pins are arranged in three intertwined layers (inner, middle, outer) of 10 pins each. Overview of the compared electrode and cap types. 3D schematic of (**c**) Flower and (**f**) Multipin electrode; Photographs of coated (**g**) Flower and (**h**) Multipin electrode; integrated into 64 channel caps—(**i**) Flower cap, and (**j**) commercial Multipin cap; Equidistant electrode layout with 64 channels, including color-coded positions of the different electrode types: (**d**) Flower electrode setup, (**e**) Multipin electrode setup.

The novel Flower electrode consisting of multiple angled pins in an intertwined arrangement on a common base plate is depicted in Fig. 1a and b. Each Flower electrode consists of 30 pins in a radial, layered structure. There are 3 circular layers consisting of 10 pins per layer. The electrode’s base diameter, pin height, and pin diameter are 12.5 mm, 5 mm, and 1 mm, respectively.

The design parameters provide mechanical stability and increase the electrode-scalp contact area when the electrode is being pressed against a surface. When the electrode is pressed to the head, the electrode pins pass through the hair and bend when in contact with the scalp, which increases the contact area. The increase in contact area not only contributes to improving electrical connection but also comfort. Furthermore, under high pressure or impact, the pin can deform until it is flattened, which further decreases the possibility of pain or skin damage due to excessive local pressure, and allows a high level of adaptivity to the head curvature.

The electrode substrate is produced by vacuum casting of flexible thermoset polyurethane (UPX 8400, Sika AG, Switzerland). The substrate is then coated with Silver/Silver-Chloride (Ag/AgCl) through a multi-phase electroless plating process^1,49^. Ag/AgCl is the gold standard for biopotential electrodes due to its electrochemical stability and has been successfully used for dry EEG electrodes before^1,4,5,25^. The substrate and the coating material have passed biocompatibility tests.

For comparison, conventional Multipin dry EEG electrodes are chosen^1,2,4,5^. The electrodes consist of 30 pins with straight pin orientation on one common baseplate (Fig. 1f) and Ag/AgCl coating (Fig. 1h). Multipin-shaped dry electrodes have been validated before and shown to provide signal quality comparable to gel-based EEG Ag/AgCl electrodes^1,2,23,25,42,47^. For the study at hand, we chose a commercial, 64-channel dry EEG cap (waveguard touch CY-261, ANT neuro B.V.) with a quasi-equidistant electrode arrangement (Fig. 1j). The cap comprises three types of Multipin electrodes with a homogeneous shore hardness of A98 and pin lengths of 1.5 mm, 3 mm, and 6 mm for head regions with different hair lengths and densities, respectively (Fig. 1e). The cap size, fabric, and cut are identical between the Multipin and Flower caps. The only differences are the electrodes and their mechanism used for fixation in the cap.

64 Flower electrodes with homogeneous pin lengths (Fig. 1c) with Ag/AgCl coating (Fig. 1g) were produced and integrated into the same fabric cap type and arrangement (Fig. 1i) by means of our recently proposed replaceable electrode system^50^. Frontal electrode positions were equipped with electrodes of shore hardness A60, while positions with dense hair were equipped with electrodes of shore A85 (Fig. 1d).

### In-vivo study design

An overall number of 20 healthy volunteers participated in the study: 8 females and 12 males. The average age of the volunteers was 25 ± 4 years. The average head circumference was 56 ± 1 cm. The hair length of female and male volunteers was 49 ± 22 cm and 5 ± 3 cm, respectively. The inclusion criteria were: age between 18 and 65 years; head circumference between 55 and 58 cm, no current or past substance abuse; no ongoing medical treatments influencing the neurological system; no skin diseases; hair type 1 (i.e. straight hair according to the Walker hair typing system^51^). We asked the volunteers to clean and dry their hair in the mornings before the study participation, to minimize the influence of possible oil, sweat, fat, and other secretion on the results. All volunteers gave written informed consent for their participation before the recordings were conducted. The study procedure is in line with the Declaration of Helsinki and was approved by the ethics committee of the Faculty of Medicine of the Friedrich-Schiller-University Jena, Germany.

The two caps were tested in a randomized order in two sessions on two separate days. A referential EEG amplifier (eego EE-225, ANT Neuro B.V., Hengelo, Netherlands) with a sampling rate of 1024 samples/second was used for all EEG recordings. For recording and review, eego software was used (eego, ANT Neuro B.V., Hengelo, Netherlands). Ground and reference electrodes were placed on the left and right mastoids, respectively, using sticker gel-based electrodes (Kendall ECG electrodes H124SG, Covidien LLC, Mansfield, UK).

Electrode and cap performance is assessed using a modified version of our established validation paradigm allowing comparison of the results to the previous studies^1,4,5^. Indications for eye blinks as well as evoked activity have been presented using a separate computer running eevoke stimulation software (eemagine Medical Imaging Solutions GmbH, Berlin, Germany). Each recording session consists of two parts: recording the EEG in a sitting position, followed by recording in supine position.

Cap application time is defined as the time starting from initial cap placement to the beginning of the first EEG recording. Accordingly, the cap preparation includes: placing the cap; placing and connecting the reference and ground electrodes; ensuring correct position and orientation of the electrodes; electrode–skin contact check and adjustment. For reference and ground electrodes, we ensured electrode–skin impedances below 50 kΩ. No impedance threshold was defined for the dry electrode positions. Instead, EEG signals on all channels were checked and the electrodes were adjusted to optimize the signal quality.

During sitting position, standard EEG recordings are done in six segments as follows: resting state with eyes open (5 min), eyes closed (5 min), triggered eyeblinks (3 min), a visual evoked potential (VEP) with checkerboard pattern reversal (3 min), resting state with eyes open (5 min), and eyes closed (5 min). Part I of the experiment was directly followed by part II which was recorded in the supine position of the volunteer and included five segments with the same sequence as in part I, excluding the VEP. During recordings in supine position, the volunteers were asked to rest the back of their head horizontally on a pillow (80 × 80 cm firm 3-chamber pillow, Sanders-Kauffmann GmbH, Bramsche, Germany).

The volunteers were asked to rank the wearing comfort of both caps. The scale for ranking was 1 to 10, with 1 being the least comfortable and 10 being the most comfortable. The scale has been assessed for its internal reliability using a robust alpha coefficient and showed excellent reliability^52^. The comfort rank was asked for both setup and at five time points during the recordings: after initial cap application, prior to the first EEG recording in sitting position (24 ± 5 min), at the end of the sitting phase (65 ± 9 min), prior to starting the supine recordings (69 ± 9 min), at the end of the supine phase (99 ± 9 min), at the end of all recordings, in sitting position (100 ± 9 min).

### Data analysis

#### Data processing

EEG and impedance acquisitions were performed using the eego software (ANT Neuro BV). The data were exported raw and subsequently analyzed with customized MATLAB scripts (MATLAB v. 2021a, The Mathworks, Natick, USA). All EEG recordings have been analyzed individually for each volunteer and electrode type.

For each recording condition except VEP, an analysis interval was manually chosen to comprise a time window of 30 s length, avoiding unwanted transient artifacts, skipping at minimum the first 10 s of the recording to allow the electrodes to stabilize, as well as keeping a minimum distance of 10 s to the end of the recording to avoid filtering artifacts. The analysis interval of the VEP recordings was defined from 10 s after the beginning until 10 s before the end of the recording.

Channel offsets were calculated as the mean over the raw data within the analysis interval. The EEG signals were then forward–backward filtered using a 30^th^-order Butterworth bandpass filter with cut-off frequencies at 1 and 40 Hz. Before filtering the VEP recordings, the first sample was subtracted from the whole analysis window to reduce possible filter artifacts.

Bad channels in the analysis intervals were visually identified by a trained operator and excluded from further analysis. Bad channels were either saturated isoelectric channels, or comprised artifactual data for more than 20% of the analyzed recording interval. For the used unipolar EEG amplifier model, isoelectric channels solely represent input voltages exceeding the dynamic range of the respective channel. Subsequently, all data were re-referenced to common average reference.

#### Impedances and channel reliability

Impedances were assessed using the integrated functions of the EEG amplifier and analyzed before the first and after the last EEG recording, with an upper threshold of 1 MΩ applied to the raw values. This threshold was set according to the manufacturer's recommendations, ensuring reliable impedance results and limiting the effects of measurement outliers.

The relative channel reliability of each channel was calculated as the number of EEG analysis intervals in which a given channel was marked as a good channel, divided by *m*, i.e. the number of all analysis intervals per electrode type in sitting (m = 100) and supine (m = 80) position. Furthermore, Spearman's rank correlation coefficient was calculated between channel groups with impedance Z > Z_T_ (with Z_T_ ranging from 10 kΩ to 1 MΩ in steps of 1 kΩ) and channel groups with channel reliability CR < CR_T_ (with CR_T_ ranging from 40 to 100% in steps of 1%).

#### Signal quality metrics

The power spectral density (PSD) was calculated using Welch's estimation method for the resting-state EEG intervals. Bad channels were interpolated by spherical spline interpolation^53^. Recordings in sitting and supine positions were analyzed separately and the repeated measurements for each resting-state condition (eyes open and eyes closed) were averaged for the respective PSD calculations. The mean alpha-band power (8–13 Hz) was calculated for the eyes-closed condition.

VEP trials contaminated with artifacts were manually identified by a trained operator and excluded from the analysis. The remaining trials were averaged with a pre-stimulus interval of 100 ms and a post-stimulus interval of 400 ms. The channel offset, calculated as the mean value of the baseline interval *t* = [− 100, − 50] ms pre-stimulus, was subtracted from each channel. Time-domain global field power (GFPt) across all channels was calculated for the averaged VEPs according to Eq. (1)^54^, where *U* is the amplitude value from the channel with index *k* or *l* out of a total of *x* channels. *x* is the number of channels after the exclusion of bad channels from the overall 64 channels.

1 $${\text{GFPt}} \, \text{=} \, \sqrt{\frac{1}{{\text{2x}}}{\sum }_{{\text{k}}= \text{1} }^{\text{x}}{\sum }_{{\text{l}}= \text{1} }^{\text{x}}{\text{(}{\text{U}}_{\text{k}}-{\text{U}}_{\text{l}}\text{)}}^{2}}$$

For quantitative comparison of the VEP traces of the Flower and Multipin electrodes, the root mean square deviation (RMSD) and Spearman's rank correlation coefficient *ρ* were calculated for each channel according to Eqs. (2) and (3). *U* is the amplitude value of data sample *i* in channel *j* for the recordings of the Flower (*Uf*) or Multipin (*Um*) electrode from a total of *n* = 513 data samples (i.e., 500 ms of data).

2 $${\text{RMSD}}_{\text{j}}\text{=}\sqrt{\frac{\sum_{\text{i=1}}^{\text{n}}{\text{(}{\text{Uf}}_{\text{ij}}-{\text{Um}}_{\text{ij}}\text{)}}^{2}}{\text{n}}}$$

3 $$\uprho _{{\text{j}}} = \frac{{\mathop \sum \nolimits_{{{\text{i}} = 1}}^{{\text{n}}} \left( {{\text{Uf}}_{{{\text{ij}}}} - \overline{{{\text{Uf}}}} } \right)\left( {{\text{Um}}_{{{\text{ij}}}} - \overline{{{\text{Um}}}} } \right)}}{{\sqrt {\mathop \sum \nolimits_{{{\text{i}} = 1}}^{{\text{n}}} \left( {{\text{Uf}}_{{{\text{ij}}}} - \overline{{{\text{Uf}}}} } \right)^{2} \mathop \sum \nolimits_{{{\text{i}} = 1}}^{{\text{n}}} \left( {{\text{Um}}_{{{\text{ij}}}} - \overline{{{\text{Um}}}} } \right)^{2} } }} $$

Further, *RMSDGFPt* and *ρGFPt* were calculated between the GFPt of both cap types for each volunteer according to Eqs. (4) and (5).

4 $${\text{RMSD}}_{\text{GFPt}}\text{=}\sqrt{\frac{\sum_{\text{i=1}}^{\text{n}}{\text{(}{\text{GFPtf}}_{\text{j}}-{\text{GFPtm}}_{\text{j}}\text{)}}^{2}}{\text{n}}}$$

5 $$\uprho _{{{\text{GFPt}}}} = \frac{{\mathop \sum \nolimits_{{{\text{i}} = 1}}^{{\text{n}}} \left( {{\text{GFPtf}}_{{\text{j}}} - \overline{{{\text{GFPtf}}}} } \right)\left( {{\text{GFPtm}}_{{\text{j}}} - \overline{{{\text{GFPtm}}}} } \right)}}{{\sqrt {\mathop \sum \nolimits_{{{\text{i}} = 1}}^{{\text{n}}} \left( {{\text{GFPtf}}_{{\text{j}}} - \overline{{{\text{GFPtf}}}} } \right)^{2} \mathop \sum \nolimits_{{{\text{i}} = 1}}^{{\text{n}}} \left( {{\text{GFPtm}}_{{\text{j}}} - \overline{{{\text{GFPtm}}}} } \right)^{2} } }} $$

Two estimates of the signal-to-noise ratio (SNR), *SNRGFPt*, and *SNRmax*, were derived. *SNRGFPt* is calculated as the ratio between the GFPt at the N75 or P100 peak and the mean value of the GFPt in the baseline interval *t* = [− 100, − 50] ms. *SNRmax* is the ratio of the N75 or P100 peak amplitude to the standard deviation of the baseline interval of the channel with maximum absolute amplitude.

#### Statistics

Tests for statistical parameter differences were performed for VEP peak latency, VEP peak power in the GFP, SNR, and the PSD for all volunteers comparing the Flower and Multipin electrode recordings. The spatial distribution of alpha band power and the VEP peak amplitudes have been tested on the level of individual channels and the level of interpolated 2D topographies. The hypothesis of a normal distribution was rejected by Kolmogorov–Smirnov tests at an alpha level of 0.05 for all aforementioned parameters. Therefore, the statistical significance of the parameter differences between VEP and SNRs was tested using a Wilcoxon signed rank test with an alpha level of 0.05. The mean PSD of the EEG bands 1–4 Hz, 4–8 Hz, 8–13 Hz, 13–30 Hz, and 30–40 Hz were tested with Wilcoxon signed rank test at a Bonferroni corrected alpha level of 0.01. Further, the effects of the two independent factors *electrode* (Flower and Multipin) and *position* (sitting and supine) on the mean PSD of the five aforementioned EEG bands were investigated by a linear mixed-effects (LME) model, with the volunteers included as random effects and the four conditions Multipin sitting, Flower sitting, Multipin supine, and Flower supine included as fixed effects. Again, the alpha level was adjusted by the Bonferroni correction.

An LME is used to test for statistical differences between comfort over time and volunteers for each of the two setups^55^. As the experimental design had multiple comfort ranks for each volunteer, the correlation of comfort among the volunteer has to be considered in the model. Wearing the caps for a longer period is assumed to lead to lower comfort. Thus, we modeled the linear relationship between comfort and time. The model consists of 10 conditions, each corresponding to one of the five time points of comfort assessment listed above and one of two cap systems. To account for the repeated measurements of participants we introduced random effects, while 10 conditions were introduced as fixed effects i.e. grouping variables. An alpha level < 0.05 is considered statistically significant.

An additional post-hoc analysis was performed. A Shapiro–Wilk test at an alpha level of 0.05 was performed to test the normality of data distribution. As not all of the sample groups were normally distributed, the nonparametric Wilcoxon matched-pairs signed rank test was used to check the statistical significance of the differences. For the Wilcoxon matched pairs signed rank tests we applied the Bonferroni correction method.

#### Electrode durability assessment

The electric function of the electrodes was tested by resistance measurements between (a) the cap connector and each electrode pin tip, and (b) the back of each electrode and the respective electrode pin tips using a Multimeter (Fluke 87 III true RMS Multimeter, Fluke AG, Everett, USA).

In addition, a mechanical durability test was performed with five Flower electrode samples, two coated and three uncoated ones. One coated and one uncoated electrode had a shore hardness of A50. One coated and two uncoated electrodes had a shore hardness of A95. Each sample was pressed 3200 times against a piece of acrylic glass, such that the pins were fully flattened on the glass surface (i.e. the pins were bent such that they were parallel to the glass surface). After the mechanical tests, all electrodes have been visually inspected for signs of mechanical wear.

## Results

### Impedances and channel reliability

**Figure 2:**
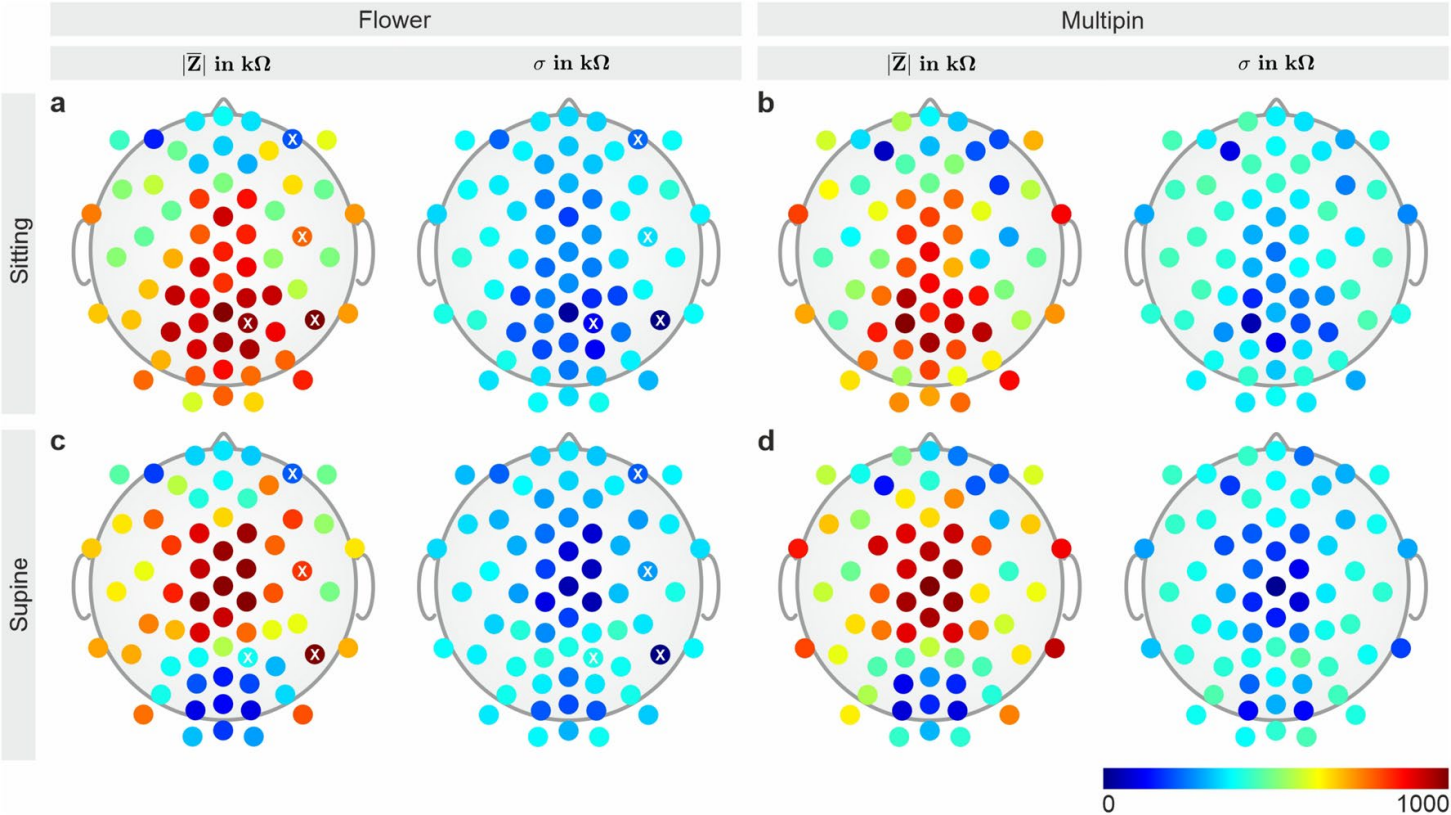
Topographic distribution of mean and standard deviation of the electrode–skin impedances. Impedances at the beginning of the EEG recordings in (**a**, **b**) sitting position, and (**c**, **d**) supine position using (**a**, **c**) the Flower electrode cap or (**b**, **d**) the Multipin electrode cap. Electrode positions that were damaged during the course of the study are marked by a white cross. The average and standard deviation of the impedance of each of the 64 electrodes was calculated over all volunteers.

The average electrode–skin impedance for the recordings in the sitting position with Multipin electrodes is 632 ± 224 kΩ at the beginning and 702 ± 219 kΩ at the end of the session. With Flower electrodes the impedances are 686 ± 217 kΩ at the beginning and 716 ± 207 kΩ at the end, respectively. For the supine position session, Multipin electrodes show an average electrode–skin impedance of 586 ± 262 kΩ at the beginning and 608 ± 263 kΩ at the end, compared to Flower electrodes with 584 ± 259 kΩ at the beginning and 593 ± 267 kΩ at the end. The corresponding topographic distributions of the impedances for both caps are depicted in Fig. 2. In the sitting position, a higher impedance level is visible for the central, parietal, and occipital areas, while the impedance at the frontal and temporal regions is lower in both caps. In the supine position, the impedance level in the occipital region is lower for both electrode types. A higher impedance level at the anterior-frontal electrodes is visible for the Multipin cap in supine position.

**Figure 3:**
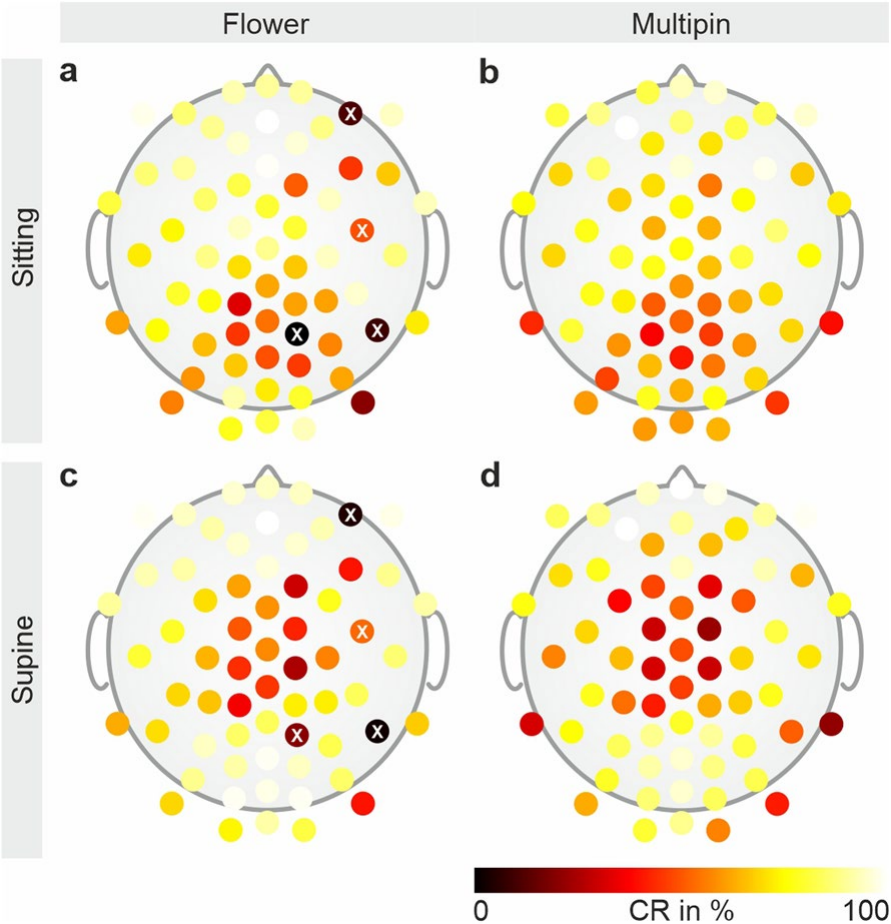
Topographic distribution of the relative channel reliability CR. Channel reliability of (**a**, **c**) the Flower, and (**b**, **d**) the Multipin electrode cap, calculated based on the bad channel evaluations for all volunteers and all EEG recordings in the (**a**, **b**) sitting and (**c**, **d**) supine position. Channel reliability was calculated as the number of EEG analysis intervals in which a given channel was marked as a good channel, divided by the number of all analysis intervals per electrode type in sitting or in supine position. Electrode positions that were damaged during the course of the study are marked by a white cross.

Four channels of the Flower electrode cap have been damaged at the electric interface during the course of the study. The damaged electrodes are indicated by a white cross in Figs. 2 and 3.

The average channel reliability achieved in sitting position for all volunteers is 70 ± 15% for the Multipin and 72 ± 23% for the Flower electrode cap, respectively. In the supine position, the average channel reliability is 69 ± 22% for the Multipin and 73 ± 24% for the Flower electrode cap. The corresponding topographic distributions of the relative channel reliability for both caps and recording positions are depicted in Fig. 3. In the sitting position, lower channel reliability is visible in the parietal area of the head for both caps. In the supine position, the area of lower channel reliability shifts towards the central region of the head.

### EEG signal quality

#### Spontaneous EEG

**Figure 4:**
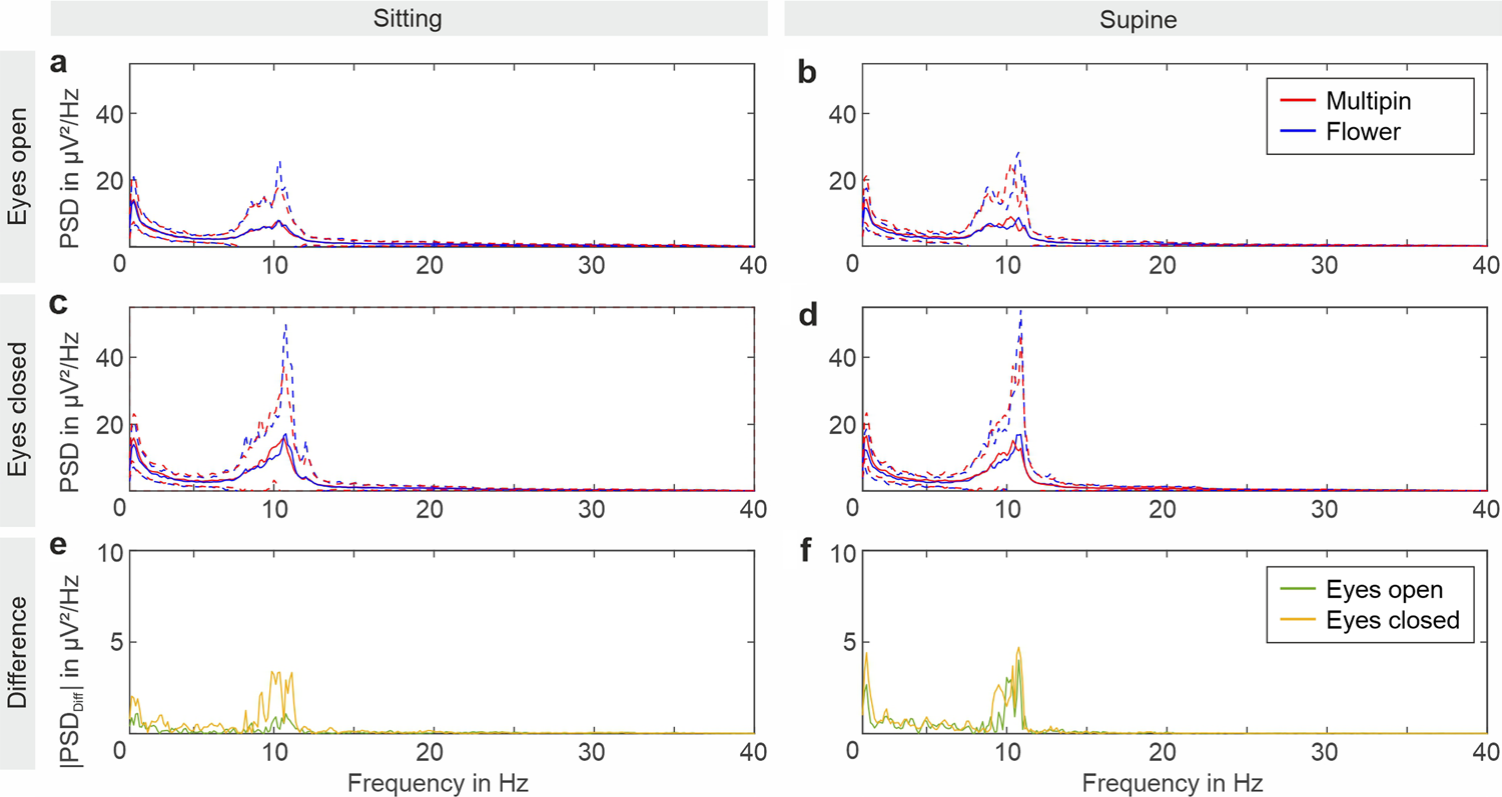
Grand average power spectral density (PSD) for the resting state activity EEG. The analyzed data were recorded with (**a**, **b**) eyes open, and (**c**, **d**) eyes closed using Flower and Multipin electrode caps in (**a**, **c**) sitting recording position and (**b**, **d**) supine recording position. The mean is indicated by solid lines and the standard deviation is indicated by dotted lines. In (**e**, **f**) the absolute value of the difference between Flower and Multipin dry electrode recordings with open or closed eyes in (**e**) sitting position and (**f**) supine position are shown. The PSD was calculated using the Welch estimation method and averaged across all volunteers and all 64 EEG channels.

Figure 4 depicts the mean and SD (standard deviation) of the Welch estimation for the PSD in resting state EEG with eyes open, and eyes closed and for the recordings in sitting and supine positions. Moreover, Fig. 4e and f show the absolute difference between the PSDs of the respective recordings performed with the Multipin and Flower electrode caps.

The PSDs exhibit increased power for the alpha band during recordings with eyes closed for both cap types in sitting and supine positions. The absolute differences between the mean PSD of the Multipin and Flower recordings are below 5 µV^2^/Hz for all conditions, i.e. all frequency bands, both sitting and supine positions, and open or closed eyes.

According to the Wilcoxon signed rank test, no significant differences are found between the PSD of both electrode types in sitting condition. Statistically significant differences between the Multipin and Flower electrode recordings in the supine position were found in the recordings with eyes open for 1–4 Hz and 4–8 Hz. Statistically significant differences between Multipin and Flower electrode recordings with eyes closed were identified for 1–4 Hz only. Individual mean and standard deviation of the power in the different EEG bands together with the corresponding *p*-values are listed in Supplementary Tables S1 and S2 for the sitting and supine positions, respectively.

**Figure 5:**
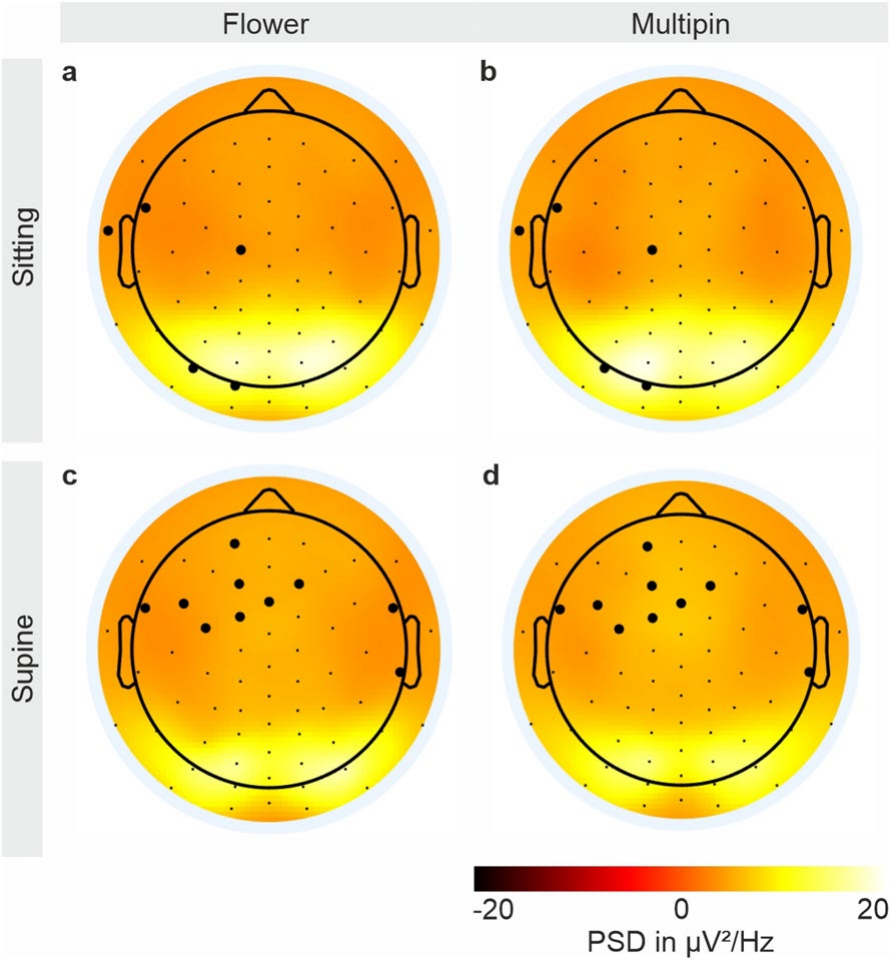
Grand average 2D interpolated topographic plots of the mean alpha band power with closed eyes. The alpha band power was calculated over all volunteers in the (**a**, **b**) sitting, and (**c**, **d**) supine position recorded using (**a**, **c**) the Flower, and (**b**, **d**) the Multipin electrode cap. Black dots indicate electrode positions. Electrode positions with statistically significant differences found by the Wilcoxon signed rank test (alpha = 0.05) are indicated by enlarged dots.

In Fig. 5, the interpolated 2D topography of the mean power of the alpha band for the recording with closed eyes, calculated over all volunteers, is shown for both caps and recording positions. The higher alpha band power in the occipital area is visible. Moreover, the alpha band power is higher in sitting condition compared to supine position.

Electrodes with statistically significant differences between recordings performed using Flower or Multipin electrodes are highlighted by enlarged black dots in Fig. 5. For the resting state EEG recordings with closed eyes in sitting position, we identified five electrodes with statistically significant differences: in the left fronto-temporal, left central, and left occipital area. For the respective recordings in supine position, an overall number of 10 channels have been identified with statistically significant differences: in the left fronto-temporal, anterior-frontal, and right temporal areas.

Differences in the EEG bands of recordings with the Multipin and Flower caps in resting conditions with eyes open and eyes closed have been analyzed by applying an LME model. The Multipin sitting position was taken as a reference condition by the model and compared to the conditions (1) Flower sitting, (2) Flower supine, and (3) Multipin supine. The intercept is statistically significant for all LME models. No significant differences were found comparing the Flower sitting position to the Multipin sitting position. The models of both the eyes open recordings and eyes closed recordings indicate significant differences in the 13–30 Hz and 30–40 Hz frequency bands comparing the recordings in supine position (both Multipin and Flower) to recordings with Multipin electrodes in sitting position. The detailed results of the LME models for the five frequency bands, and the four conditions (Multipin sitting, Flower sitting, Multipin supine, and Flower supine) are shown in Supplementary Tables S3 and S4 for the resting state eyes open and eyes closed, respectively.

#### Evoked activity

**Figure 6:**
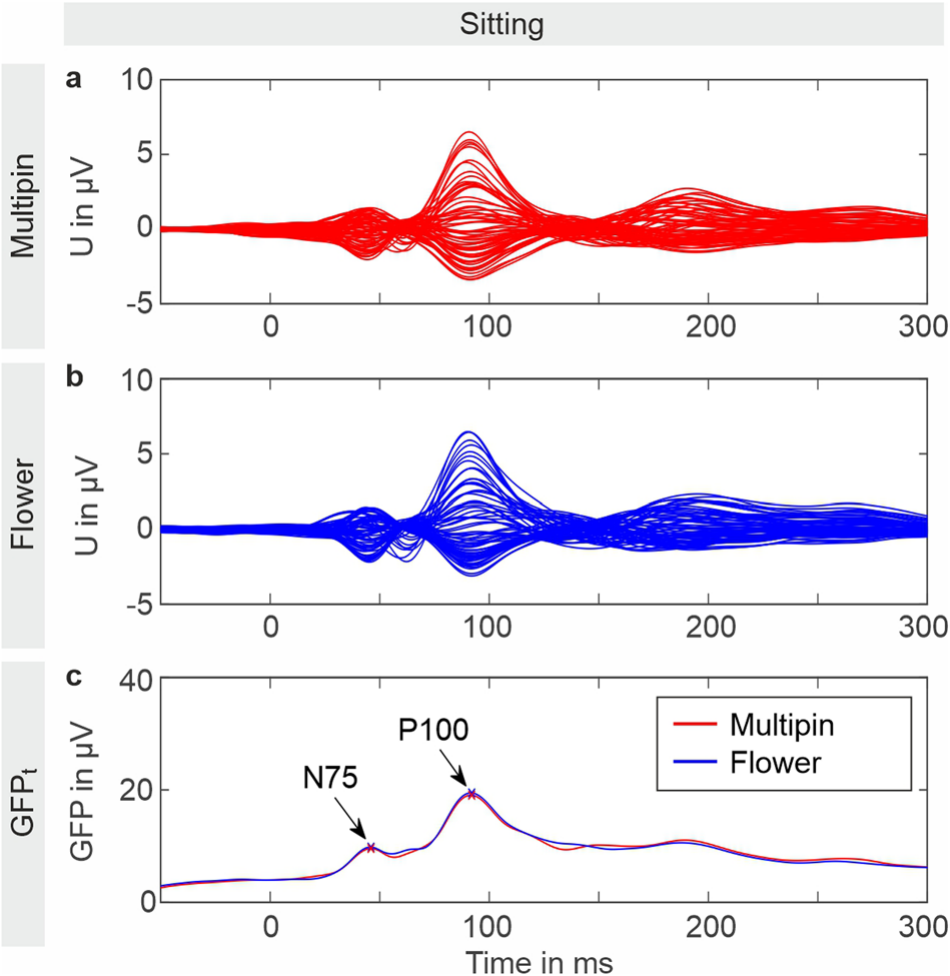
Grand average VEPs and GFPt over all volunteers. VEP butterfly plots showing all channels of (**a**) Multipin electrodes and (**b**) Flower electrodes. Plot (**c**) shows the GFPt of Flower and Multipin electrodes with marked N75 and P100 peaks.

Figure 6 shows the grand average VEP calculated over all volunteers for a period of 50 ms pre-stimulus and 300 ms post-stimulus. The butterfly plots of all channels for the Flower and Multipin electrodes (Fig. 6a and b) as well as the overlay plot of the GFPt show similar results for both electrode types. The grand average GFPt amplitudes are 9.8 µV and 9.6 µV at the N75 peak and 19.5 µV and 19.1 µV at the P100 peak for Flower and Multipin electrodes, respectively. The grand average N75 and P100 peak latencies are 46 ms and 92 ms and do not differ between electrode types.

The grand average of the RMSD calculated between individual corresponding channels of Flower and Multipin is 0.87 ± 0.18 µV. The grand average of the individual channel's correlation coefficient is 0.6 ± 0.07. The RMSD and correlation coefficients of the GFPt between both electrode types over all volunteers are 2.73 ± 1.11 µV and 0.81 ± 0.14, respectively. No statistical differences between Flower and Multipin electrodes were found for the GFPt at the N75 and P100 peaks for amplitudes (${\text{p}}_{\text{N75}}\text{= 0.91}$ and ${\text{p}}_{\text{P100}}\text{= 0.68}$) or latencies (${\text{p}}_{\text{N75}}\text{= 0.86}$ and ${\text{p}}_{\text{P100}}\text{= 0.59}$).

The grand average SNRGFPt values of the N75 and P100 peaks are 6.8 ± 3.1 and 11.8 ± 4.8 for the Multipin electrode, and 6.8 ± 3.6 and 12.3 ± 6.7 for the Flower electrode cap. The grand average SNRmax of N75 and P100 are 13.0 ± 9.6 and 25.0 ± 13.7 for the Multipin electrodes, and 11.9 ± 8.5 and 23.6 ± 10.5 for the Flower electrodes. No significant differences were found between the Flower and Multipin electrodes at those main peaks for the SNRGFPt (${\text{p}}_{\text{N75}}\text{= 0.79}$ and ${\text{p}}_{\text{P100}}\text{= 0.88}$) or the SNRmax. (${\text{p}}_{\text{N75}}\text{= 0.41}$ and ${\text{p}}_{\text{P100}}\text{= 0.77}$).

**Figure 7:**
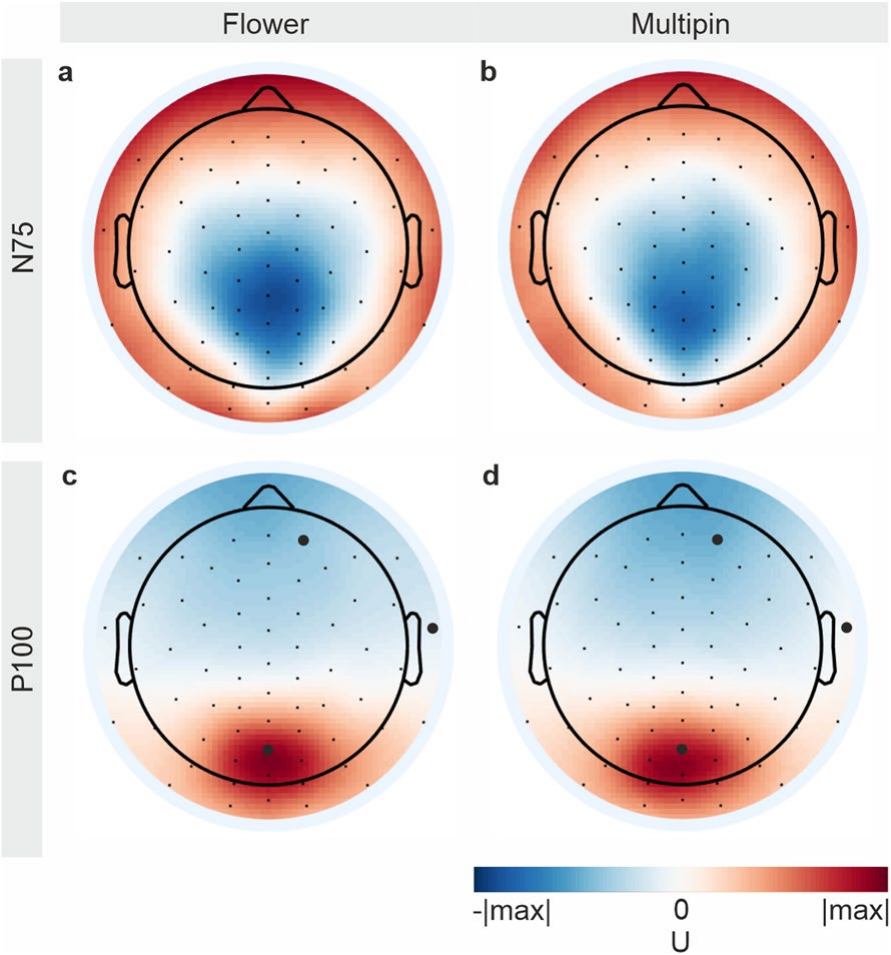
Grand average 2D topographic plots of the visual evoked potentials (VEP) calculated over all volunteers. Interpolated plots for the (**a**, **c**) Flower and (**b**, **d**) Multipin electrode recordings of (**a**, **b**) the N75, and (**c**, **d**) the P100 peak. Black dots indicate electrode positions. The enlarged black dots are indicating significantly different VEP channels found by the Wilcoxon signed rank test (alpha = 0.05).

A comparison of the VEP topographies is shown in Fig. 7a for the N75 peak and Fig. 7b for the P100 peak. No significant differences were found between the individual channels of the two electrode types at N75. Three channels differ significantly at the P100 peak, indicated by enlarged black dots in Fig. 7.

### Applicability and durability

#### Preparation and recording times

The average preparation times were 23 ± 5 min for the Multipin electrode cap and 24 ± 6 min for the Flower electrode cap.

The total recording times for the sitting position were 65 ± 9 min and 66 ± 8 min with Multipin and Flower setup, respectively. The total recording times for the supine position were 30 ± 2 min and 32 ± 3 min with Multipin and Flower setup, respectively.

#### Perceived comfort

The subjective wearing comfort reported by the volunteers right after the initial cap application was 7.4 ± 1.4 for the Multipin and 8.7 ± 1.1 for the Flower electrode cap. The comfort decreased towards the end of the sitting session and was ranked 4.9 ± 2.3 and 8.2 ± 1.3 for Multipin and Flower cap after 65 min, respectively. In supine position, the comfort further decreased compared to the sitting position. At the beginning of the recordings in supine position, the comfort ranking was 5.1 ± 1.3 and 6.1 ± 1.6 for Multipin vs. Flower electrodes. At the end of the supine position recordings (after 34 min in supine position, overall 99 min of cap wearing), the reported comfort had further decreased to 3.1 ± 1.7 and 4.9 ± 1.7. Returning to sitting position, the average reported comfort increased again and was 5.2 ± 2 and 7.2 ± 1.8, for Multipin and Flower respectively (at the end of the entire paradigm).

**Figure 8:**
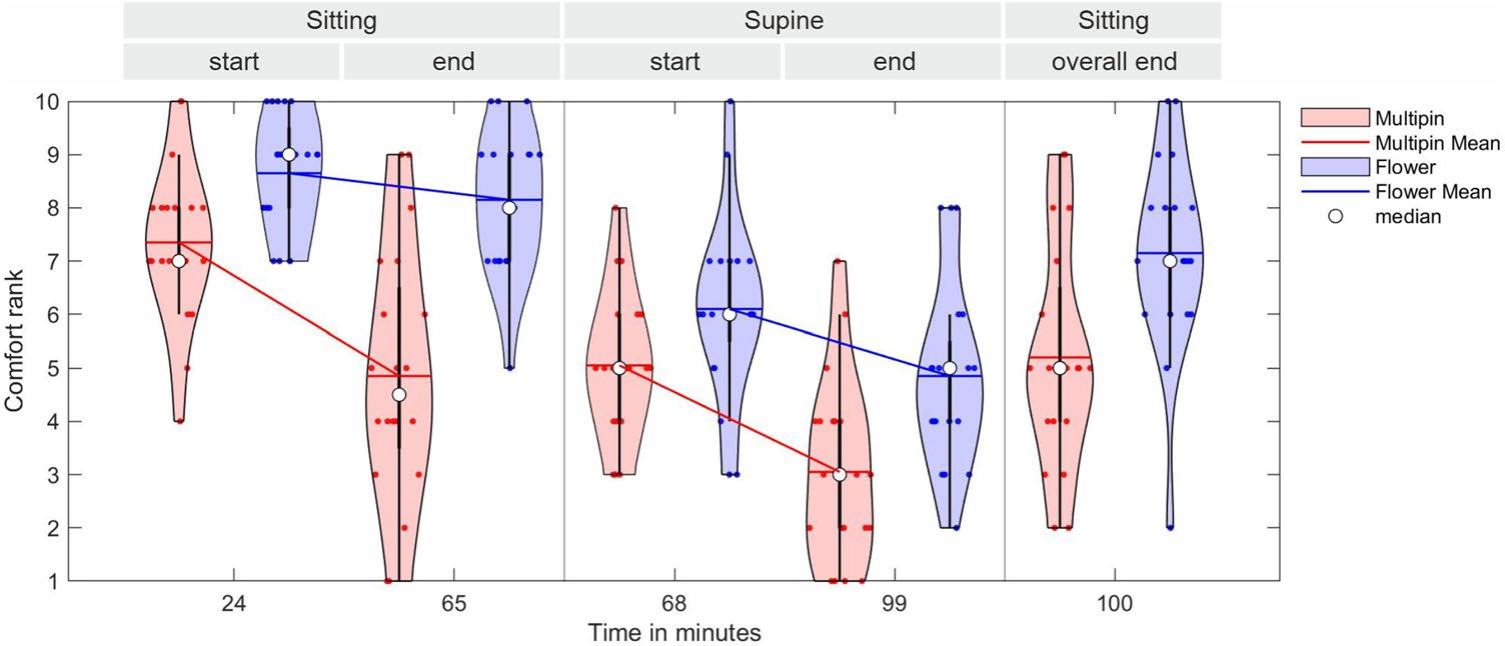
Violin plot of the wearing comfort ranking distribution for both electrode types and both body positions. Volunteers reported their perceived comfort at an overall number of 5 time points during the recordings: after initial cap application, at the end of the sitting recordings, at the beginning and end of the supine recordings, and at the overall end of the recordings (returned to sitting position). Indicated wearing times are grand averages calculated over all volunteers and both caps. For each violin plot, the mean comfort score is shown with horizontal solid lines, while the median is shown with a white dot. Individual results are indicated by colored dots.

Figure 8 shows violin plot distributions of the volunteers’ reported comfort ranking at each of the five conditions for both of the systems, Multipin (red) and Flower (blue).

Using the initial comfort of the Multipin electrodes (at 24 min) as a reference, the fixed effect coefficients indicate (1) a significantly increased comfort with Flower electrodes both at 24 min and 65 min, (2) No significant difference for Flower electrodes after 100 min, (3) a significant decrease of comfort for Multipin electrodes after 65 min and 100 min, (4) a significant decrease for both electrode types in supine position at 68 and 99 min. Detailed results of the mixed effects model for the comfort ranking are available in Supplementary Table S5.

The post-hoc Wilcoxon matched-pairs signed rank test at an alpha level of 0.01 showed a significant difference in wearing comfort between Multipin and Flower electrodes at each stage of the experiment. Already at the beginning of the measurements, the comfort of the Flower cap was significantly higher than that of the Multipin cap (*p* = 0.0007). At the end of the sitting session, the Flower electrodes were significantly more comfortable than the Multipin again (*p* = 0.0002). The same observation holds for the supine session at the end (*p* = 0.0037) as well as at the overall end of the measurements after returning to sitting position (*p* = 0.0008).

#### Electrode durability

Electrical resistance measurements of all Flower electrodes showed pin-to-backside resistance below 10 Ω before and after the measurements on 20 volunteers (20 cap applications and removals, 34 measurement hours overall). However, after the measurements, the pin-to-connector resistance of 2 channels (4RC, 4LB) showed unstable and high resistance connections > 1 MΩ. The cause of the high resistance was broken solder connections.

The mechanical durability tests resulted in no signs of mechanical changes in the PU base material, especially no signs of cracks or clefts at the pins nor at the link of the pins to the base plate. We observed signs of uncritical wear on the coating (i.e. polished, shiny pin tips) similar to the previous findings following extended electrode use^4^. The electrodes were still well conductive with resistance values < 2 Ω (measured pin-to-back).

## Discussion

We developed and successfully validated the performance of a novel 64-channel Flower electrode cap for comfortable dry EEG acquisition. For this purpose, we compared the novel Flower electrodes with our commercially available previous generation of Multipin dry electrodes^1^. These Multipin electrodes have been studied and validated for different applications and setups. In each of these previous studies, the signal characteristics of Multipin electrodes were reported equivalent to those of conventional gel-based electrodes in the sitting position^1,2,4,5^. In the study at hand, we provided evidence for Flower and Multipin electrodes to be equivalent in terms of applicability, reliability, durability, and signal characteristics. The different electrode fixations in the Flower and Multipin caps result in an increased electrode height for the Flower cap, eventually increasing electrode adduction slightly. Despite this, the Flower electrodes have demonstrated significantly increased wearing comfort compared to Multipin electrodes. The specific design of the Flower electrodes and the increased wearing comfort allowed—for the first time—to additionally perform validation of comfort, channel reliability, and signal quality for recordings in supine position.

Beyond the study at hand, comparing Multipin and Flower dry electrodes, the used cap type, testing paradigm, and comparison metrics are identical not only for the Multipin and Flower caps, but also for commercial gel-based caps used in previous studies. Consequently, the signal characteristics, reliability, and impedance metrics can be interpreted and compared to gel-based electrode cap performance as well^1,2,4,5^. The used comfort ranking scale differed in the study at hand compared to previous studies. However, from a qualitative point of view, the comfort increase observed for the Flower electrodes is at the order of magnitude of the gel-based electrode comfort reported previously^2^.

### Preparation

During the placement of the caps, dry electrodes tend to tilt and thus might need manual correction such that they are placed perpendicular to the scalp. The two operators consistently reported that the Flower electrodes are easier to recognize when tilted as well as partly easier to grip and adjust compared with the Multipin electrodes. This might be explained partly by the different geometry of the electrodes and partly by the different electrode holding mechanism. One should consider two different approaches for the adjustment of the two types of electrodes: Flower electrodes might need rubbing and up/down movements to better get through the hair while Multipin electrodes might need sideward movements (brushing) to better get through the hair. All these movements are typically small and depend on the hair characteristics of the volunteer. Experienced volunteers can perform these movements easily themselves. Feedback from the volunteer supports the electrode adjustment by the operator. Overall, the two operators reported that the Flower electrode's geometry and flexibility help in the adjustments and thus is easier to adjust compared with the Multipin electrodes. Visual assessment after the recordings confirmed the success of the adjustments for most electrodes since most skin marks were symmetrically showing all pins.

The reported average preparation time of 24 ± 5 min is considerably longer than in previous Multipin dry electrode studies, but still significantly reduced compared to the previously reported preparation time for a gel-based cap with 64 channel layout (39 ± 18 min)^2^. An influence of operator experience was reported^5^ and was evident in the results of the study at hand. Consequently, a reduced dry electrode preparation time can be expected for experienced operators and users.

### Wearing comfort

During recordings in sitting position, the Flower electrode maintained an average comfort within the top 20% of the range even after 65 min of wearing. In contrast, the comfort of Multipin electrodes significantly decreased during this period to an average of 50% of the range. A decrease in wearing comfort over time was reported in previous publications for Multipin type electrodes. The comfort of Flower electrodes, therefore, is superior to Multipin electrodes with shore hardness A98, to Multipin with reduced shore hardness^25^, to arch-shaped electrodes^31^, and to spring-loaded Multipin electrodes^24,32^. Only Di Fronso et al. reported increased comfort with Multipin electrodes over time. However, this was hypothesized to be related to the specific paradigm of the study which may distract from the EEG cap wearing comfort^2^. A detailed overview of the comfort ranking statistics reported in the aforementioned studies is provided in Supplementary Table S6.

In the supine position, volunteers reported higher comfort with the Flower electrodes (average above 50% of the range) compared to Multipin electrodes. However, still, the comfort levels are lower compared to the sitting position. The average comfort of Multipin electrodes in supine position started at 50% of the range and further decreased to 30% during wearing time. This indicates that Multipin electrodes are unsuitable for recordings in supine position, except for emergency applications, where comfort may be a minor concern. Interestingly, volunteers reported that they were feeling the base of the electrodes and experienced them as painful while the pins themselves were not painful. We, therefore, hypothesize that the lower comfort is caused by the electrode fixation mechanism made from hard polymer and can thus be further increased by optimization of the electrode assembly, specifically the electrode holder design and overall thickness.

Volunteers reported lower comfort in the frontal head region compared to all other regions in the sitting position. These observations are in line with earlier studies of the Multipin electrodes^1,4^. The main reasons are the lower pain thresholds in the frontal head region^56^ and the higher pressure at the circumferential electrodes due to the cut of the cap textile^47^. The Flower electrodes had an improved comfort compared to the Multipin electrodes, the latter explicitly reported uncomfortable by some volunteers. These findings are supported also by less deep and less visible skin marks of the Flower electrodes on most volunteers. The reduced shore hardness of the Flower electrodes also contributed to the reduction of skin marks. Other than temporary visual marks, no skin irritation effects were reported by any volunteer.

### Impedance and channel reliability

The mean impedance over all channels of the Flower electrodes is increased by 5.07% in the sitting position but decreased by 1.45% in the supine position compared to the Multipin electrodes. In addition, the mean impedance change over all channels was considerably lower for the Flower (3.04%) than for the Multipin electrodes (7.42%). Moreover, the impedances were more stable in the supine position compared to the sitting position. This observation may support two assumptions: Due to the higher adaptivity of the Flower electrode design, the electrodes establish a more stable contact with the skin compared to the less flexible Multipin electrodes. Moreover, the increased adduction at occipital positions during the supine position leads to lower and more stable impedances compared to the sitting position, which is in line with the results of Fiedler et al.^47^.

Increased impedance values in the central and parietal head regions have been found for both electrode types. Similar regions of increased interfacial impedance and reduced channel reliability have been reported in previous publications^1,4^ and are known to result from reduced adduction force exerted by the cap fabric at central regions of the head compared to the circumferential electrode positions^4^. The reduced contact pressure causes increased interfacial impedance and reduced contact stability^47^.

The Multipin cap’s average impedance of 632.19 kΩ at the beginning and 701.50 kΩ at the end of the recording is notably increased and the channel reliability of 70% in the sitting position is lower than in previous publications^1,4^. Differences with respect to previous publications may be related to operator experience^5^. While dry electrode impedance may often decrease over time due to sweat or skin hydration effects, it also strongly depends on interface stability (e.g. movements), cap type, and environmental conditions (e.g. room humidity and temperature) which will strongly influence the sweat layer build-up likelihood and speed. The specific fabric of the compared EEG caps, being air-permeable, in addition to the overall strong movements at the electrode–skin interface during the change of body posture (sitting vs. supine position) and respective adduction changes (sitting without head rest vs. pillow during supine position) may have caused the observed increase of the impedances.

The Flower electrodes showed slightly higher channel reliability than the Multipin electrodes both in sitting and supine positions. During the study, solder connections of four channels of the Flower cap were damaged, which are responsible for 4% of all non-operational Flower electrodes in both positions. Consequently, with fully functional electrodes, the channel reliability of the Flower electrodes would have been further increased.

No considerable differences in global channel reliability were found comparing sitting and supine positions. The topographic shifts in both impedance and channel reliability are caused by changes in the cap adduction due to the different head positions and mechanic support. During sitting, the head was in an upright position without head rest. In supine position, the head was rested in a horizontal position on the pillow.

The Ag/AgCl coating procedure used during the production of the Flower electrodes remains unchanged compared to the one used previously for the Multipin electrodes. We, therefore, expect a similar electrical durability as reported by Fiedler et al.^4^. Following a dedicated wearing test, the study reported that the coating does not show significant degradation after 800 applications of the electrodes^4^.

### Signal quality in sitting position

General signal characteristics for both electrode types are in line with previous publications^1,2,4,5^ for the sitting position. The pairwise statistical comparisons and the LME models of the Multipin and Flower recordings mean PSDs found no significant differences in any of the EEG bands neither with open nor closed eyes.

The 2D topographies of the mean alpha band PSDs show the expected increased power in the occipital regions for both electrode caps in the sitting positions. The five channels showing significant differences are located primarily in the left hemisphere and close to the rim of the cap, near facial and neck muscles. These significant differences might therefore be related to (1) a less stable contact of electrodes close to the cap rim, and (2) differences in muscle tension and activity during the recordings. We did not observe a correlation between significantly differing channels and channels with high impedance values. This again is in line with previous findings, that high electrode–skin impedance is not directly related to signal quality in dry electrodes^4^.

Overall, no systematic differences in the analysis of the spectral range from 1 to 40 Hz were found between Multipin and Flower electrode recordings in the sitting position. Therefore, the Flower electrodes are assumed to perform equivalent to Multipin dry electrodes in terms of signal quality. Given our previous studies, this finding implies that Flower electrodes will provide equivalent signal quality to gel-based electrode recordings in the same frequency range.

The mean RMSD values and Spearman’s rank correlation coefficients calculated between VEPs recorded with both electrode types are similar to the values found in^1,4^. This observation is valid both for the RMSD and correlation calculated for individual channels as well as when calculated for the GFPt. The equivalent results imply that Multipin and Flower electrodes perform equally well compared to gel-based electrodes. The mean correlation coefficient of > 0.8 for the GFPt shows a high correlation between the VEP time courses of both cap types and is within the order of magnitude of intra-individual variability^57^. No significant differences were found between the N75 and P100 peak amplitudes and latencies as well as for both types of SNR estimations at those peaks. Significant differences between the mean GFPt of the individual channels were found only at the P100 peak for three channels, mainly close to the rim of the cap (Fig. 7). This is in line with the results of Fiedler et al.^4^. No systematic differences in the time domain analysis of peak amplitudes, latencies, and topographies of the checkerboard reversal VEP were found.

### Signal quality in supine position

For the supine position, the pairwise comparison of Multipin and Flower recordings mean PSDs showed significant differences for the lower frequency bands of 1–4 Hz and 4–8 Hz, for eyes opened and in the frequency range 1–4 Hz for eyes closed. Here, the Multipin recordings show a higher mean power and SD compared to the Flower electrodes. This could be related to the lower adaptivity and reduced wearing comfort of the Multipin cap in the supine position, which may have caused low-frequency changes in the electrode–skin interface, i.e. increased sweating as well as electric potential changes caused by skin stretching, skin compression or movement^58–60^. The same effect may also explain the significantly increased spectral power in the 4–8 Hz range of the Multipin electrode recordings during supine condition found in the LME model of the recordings with eyes open. In general, the observation of amplitude and thus spectral power dependencies related to head position have been reported for gel-based EEG recordings as well, and may be further related to changes in the thickness of the cerebrospinal fluid (CSF)^61^.

A slightly lower mean alpha band power is evident for both electrode types in the supine position compared to the sitting condition. This may be caused, again, by reduced comfort, as the alpha activity tends to decrease when the volunteer is engaged in mental concentration or physical movement and becomes tense, apprehensive, or anxious^62^. A contrary effect is the generally less engaged postural muscles holding the head in the supine position, leading to reduced power in the higher frequency bands (> 20 Hz)^3,63^. This assumption is in line with the significantly lower estimates of both Flower and Multipin recordings in the supine position compared to Multipin sitting of the LME models of 13–30 Hz and 30–40 Hz. In addition to the relaxation of the postural muscles, the higher wearing comfort of the Flower electrode cap might allow volunteers to relax better. The high sensitivity of alpha and delta band power to body position and selective focus was reported to be related to cortical inhibition and altered sensory and cognitive processing^64,65^.

In the supine position, the alpha band power of the Multipin cap in the frontal regions is increased relative to the Flower electrode as well as to recordings in sitting condition. This is leading to significant differences between the mean alpha band PSDs of the Flower and Multipin recordings in ten channels (Fig. 5). The increased power might be due to artifact residues and bad channel interpolation due to a loose cap fit and hence less stable contact in the frontal electrodes, observed with the Multipin cap in supine position. In line with these observations, the Multipin cap’s impedance values of eight of the significantly deviating channels are considerably increased compared to the sitting position. Moreover, for the Flower cap in supine position, seven of the significant channels show lower impedances than in the Multipin cap. This may indicate a better fit and thus applicability of the Flower electrode cap for EEG recordings in the supine position. Future studies may extend the systematic investigation of the effects of movement artifacts, wearing comfort, and cap adduction in different regions of the head.

### Outlook

This study included 20 healthy adult volunteers with hair type 1 according to the Walker hair typing system (i.e. straight hair)^51^. Future studies will involve a larger sample group, and add distinct studies for more diverse hair types. For this purpose, adapted electrode pin length and flexibility in combination with the replaceable electrode fixation mechanism will allow individualized cap fit and consequently both further increased channel reliability and wearing comfort. The replaceable electrode mechanism will be optimized, to further increase the comfort specifically for recordings in supine position. Moreover, while the current study used the same frequency band (1–40 Hz) as previous publications^2,4,5,31,48,49^, future studies may investigate the use of dry Flower electrodes for extended frequency bands including DC-EEG or high-frequency oscillations.

The effect of operator training and experience has previously been shown for Multipin electrodes in a multicenter study^5^ and can be assumed to be an important influence when investigating the performance of novel electrode types. Consequently, the observed performance differences both within the operators of the current study and comparing the results to previous studies may be further investigated in a larger group of operators and EEG centers.

For the first time, the Flower electrodes allowed comfortable dry recordings in supine position. In future studies, we will investigate their use for dry high-density EEG with sensitive populations, in sleep^37–41^ and emergency applications^9^, and recordings with infants and neonates^42–45^. Mechanical impact on skin and head as well as the resulting wearing comfort have an even higher impact in these sensitive populations and the acceptance of home-side EEG recordings for clinical applications^24^. The significantly increased comfort of the Flower electrodes compared to conventional Multipin electrodes consequently will play an important role in the use of dry electrodes for rapid EEG recordings in these fields of application.

## Conclusions

We present a novel Flower electrode cap that has been successfully validated in an in-vivo study on 20 healthy volunteers. The results prove the equivalent performance of the novel Flower electrodes compared to our previous Multipin dry electrodes in terms of preparation time, electrode–skin impedance, channel reliability, and signal quality, thereby implying equivalence to gel-based electrodes. Most importantly, while maintaining the aforementioned performance, the wearing comfort was significantly outperforming the Multipin electrodes. The increased comfort not only allows considerably longer dry EEG recordings but—for the first time—dry EEG recordings in supine position. This will contribute to extending the fields of application for dry EEG in sensitive populations, as well as in sleep and multimodal data acquisitions.

### Supplementary Information

Supplementary Tables.

## Data Availability

The data are available upon reasonable request to the corresponding author.
