# Supplementary material for: Flower electrodes for comfortable dry electroencephalography
Source: Sci Rep. 2023 Oct 3;13:16589. doi: 10.1038/s41598-023-42732-8 (PMC10547758; doi:10.1038/s41598-023-42732-8)
Supplement: Supplementary file 1 — Supplementary Tables. [file 41598_2023_42732_MOESM1_ESM.pdf]

Flower electrodes for comfortable dry electroencephalography

Indhika Fauzhan Warsito[1,+], *Milana Komosar*[1,+], *Maria Anne Bernhard*[1],

Patrique Fiedler[1], *and Jens Haueisen*[1,2]*

[1] Institute of Biomedical Engineering and Informatics at the Technische Universität Ilmenau, Ilmenau, Germany

[2] Department of Neurology, Biomagnetic Center, University Hospital Jena, Jena, Germany

[+] First and second author indicate equal contributions to the paper.

* corresponding author:

Jens Haueisen

email: jens.haueisen@tu-ilmenau.de

ORCID

Indhika Fauzhan Warsito – 0000-0003-0498-9867

Milana Komosar – 0000-0002-2223-0224

Maria Anne Bernhard – 0009-0007-6959-319X

Patrique Fiedler – 0000-0001-9196-0717

Jens Haueisen – 0000-0003-3871-2890

Supplementary information

Supplementary Table S1 Grand average spectral power in the individual EEG bands calculated over all volunteers and channels. Results are reported together with the corresponding p-values of the Wilcoxon signed rank test between Multipin and Flower recordings with open and closed eyes in the sitting position.

EEG band	Cap	Eyes open		Eyes closed	
		Band power $[\mu V^2/Hz]$	p-value	Band power $\mu V^2/Hz$	p-value
1-4 Hz	Multipin	6.10 ± 2.03	>0.999	7.25 ± 2.62	0.0620
	Flower	6.07 ± 2.19		6.48 ± 2.56	
4-8 Hz	Multipin	2.51 ± 1.18	0.9405	3.31 ± 1.84	0.1672
	Flower	2.46 ± 1.27		2.98 ± 1.76	
8-13 Hz	Multipin	4.19 ± 3.98	0.6813	7.70 ± 6.07	0.3135
	Flower	4.25 ± 4.59		7.40 ± 7.01	
13-30 Hz	Multipin	0.69 ± 0.49	0.1560	0.75 ± 0.45	0.0522
	Flower	0.64 ± 0.49		0.60 ± 0.51	
30-40 Hz	Multipin	0.28 ± 0.15	0.2959	0.24 ± 0.11	0.1354
	Flower	0.25 ± 0.13		0.21 ± 0.08	

Supplementary Table S2 Grand average spectral power in the individual EEG bands calculated over all volunteers and channels. Results are reported together with the corresponding p-values of the Wilcoxon signed rank test between Multipin and Flower recordings with open and closed eyes in the supine position.

EEG band	Cap	Eyes open		Eyes closed	
		Band power $[\mu V^2/Hz]$	p-value	Band power $\mu V^2/Hz$	p-value
1-4 Hz	Multipin	6.15 ± 2.14	0.0090	6.90 ± 2.13	0.0013
	Flower	5.36 ± 1.98		5.72 ± 2.24	
4-8 Hz	Multipin	2.88 ± 1.41	0.0028	3.28 ± 1.90	0.0930
	Flower	2.48 ± 1.28		2.84 ± 1.53	
8-13 Hz	Multipin	4.73 ± 5.05	0.9702	7.01 ± 5.79	0.3905
	Flower	4.59 ± 5.07		6.80 ± 6.25	
13-30 Hz	Multipin	0.57 ± 0.39	0.0400	0.63 ± 0.35	0.2180
	Flower	0.55 ± 0.42		0.61 ± 0.44	
30-40 Hz	Multipin	0.18 ± 0.07	0.6813	0.17 ± 0.06	0.4553
	Flower	0.18 ± 0.09		0.17 ± 0.10	

Supplementary Table S3 Results of the linear mixed effects model comparing recordings with eyes open. SE: Standard Error, t statistics, DF: degrees of freedom, and CI*: Bonferroni corrected confidence interval.

Freq.	LME Results							
1-4 Hz		Estimate	SE	t statistics	DF	p-Value	Lower CI*	Upper CI*
	Intercept	6.0991	0.4429	13.7700	156	<0.0001	4.9441	7.2541
	Flower Sitting	-0.0322	0.2659	-0.1209	156	0.9039	-0.7257	0.6614
	Multipin Supine	0.0505	0.2659	0.1898	156	0.8497	-0.6430	0.7440
	Flower Supine	-0.7428	0.2659	-2.7932	156	0.0059	-1.4363	-0.0493
4-8 Hz		Estimate	SE	t statistics	DF	p-Value	Lower CI*	Upper CI*
	Intercept	2.5098	0.2757	9.1020	156	<0.0001	1.7908	3.2289
	Flower Sitting	-0.0487	0.1271	-0.3828	156	0.7024	-0.3802	0.2828
	Multipin Supine	0.3652	0.1271	2.8727	156	0.0046	0.0337	0.6967
	Flower Supine	-0.0301	0.1271	-0.2369	156	0.8130	-0.3616	0.3014
8-13 Hz		Estimate	SE	t statistics	DF	p-Value	Lower CI*	Upper CI*
	Intercept	4.1934	1.0390	4.0359	156	0.0001	1.4839	6.9029
	Flower Sitting	0.0576	0.4726	0.1219	156	0.9032	-1.1748	1.2900
	Multipin Supine	0.5390	0.4726	1.1406	156	0.2558	-0.6934	1.7714
	Flower Supine	0.3983	0.4726	0.8428	156	0.4006	-0.8341	1.6307
13-30 Hz		Estimate	SE	t statistics	DF	p-Value	Lower CI*	Upper CI*
	Intercept	0.6923	0.0971	7.1268	156	<0.0001	0.4390	0.9457
	Flower Sitting	-0.0492	0.0320	-1.5401	156	0.1256	-0.1325	0.0341
	Multipin Supine	-0.1222	0.0320	-3.8241	156	0.0002	-0.2055	-0.0389
	Flower Supine	-0.1456	0.0320	-4.5567	156	<0.0001	-0.2289	-0.0623
30-40 Hz		Estimate	SE	t statistics	DF	p-Value	Lower CI*	Upper CI*
	Intercept	0.2815	0.0240	11.7299	156	<0.0001	0.2189	0.3440
	Flower Sitting	-0.0304	0.0179	-1.6943	156	0.0922	-0.0771	0.0164
	Multipin Supine	-0.1066	0.0179	-5.9442	156	<0.0001	-0.1533	-0.0598
	Flower Supine	-0.1056	0.0179	-5.8876	156	<0.0001	-0.1523	-0.0588

Supplementary Table S4 Results of the linear mixed effects model comparing recordings with eyes closed. SE: Standard Error, t statistics, DF: degrees of freedom, and CI*: Bonferroni corrected confidence interval.

Freq.	LME Results							
1-4 Hz		Estimate	SE	t statistics	DF	p-Value	Lower CI*	Upper CI*
	Intercept	7.2449	0.5179	13.9885	156	<0.0001	5.8943	8.5954
	Flower Sitting	-0.7704	0.2957	-2.6051	156	0.0101	-1.5415	0.0008
	Multipin Supine	-0.3451	0.2957	-1.1670	156	0.2450	-1.1162	0.4260
	Flower Supine	-1.5205	0.2957	-5.1416	156	<0.0001	-2.2916	-0.7493
4-8 Hz		Estimate	SE	t statistics	DF	p-Value	Lower CI*	Upper CI*
	Intercept	3.3118	0.3785	8.7493	156	<0.0001	2.3247	4.2988
	Flower Sitting	-0.3282	0.1683	-1.9497	156	0.0530	-0.7672	0.1108
	Multipin Supine	-0.0295	0.1683	-0.1755	156	0.8609	-0.4685	0.4094
	Flower Supine	-0.4707	0.1683	-2.7961	156	0.0058	-0.9096	-0.0317
8-13 Hz		Estimate	SE	t statistics	DF	p-Value	Lower CI*	Upper CI*
	Intercept	7.7029	1.3639	5.6479	156	<0.0001	4.1464	11.2595
	Flower Sitting	-0.3072	0.4247	-0.7234	156	0.4705	-1.4148	0.8003
	Multipin Supine	-0.6891	0.4247	-1.6224	156	0.1067	-1.7966	0.4185
	Flower Supine	-0.9055	0.4247	-2.1319	156	0.0346	-2.0130	0.2021
13-30 Hz		Estimate	SE	t statistics	DF	p-Value	Lower CI*	Upper CI*
	Intercept	0.7516	0.0958	7.8486	156	<0.0001	0.5019	1.0013
	Flower Sitting	-0.0647	0.0301	-2.1486	156	0.0332	-0.1432	0.0138
	Multipin Supine	-0.1216	0.0301	-4.0378	156	0.0001	-0.2001	-0.0431
	Flower Supine	-0.1442	0.0301	-4.7888	156	<0.0001	-0.2227	-0.0657
30-40 Hz		Estimate	SE	t statistics	DF	p-Value	Lower CI*	Upper CI*
	Intercept	0.2403	0.0186	12.8918	156	<0.0001	0.1917	0.2889
	Flower Sitting	-0.0279	0.0144	-1.9354	156	0.0547	-0.0655	0.0097
	Multipin Supine	-0.0699	0.0144	-4.8533	156	<0.0001	-0.1075	-0.0324
	Flower Supine	-0.0698	0.0144	-4.8482	156	<0.0001	-0.1074	-0.0323

Supplementary Table S5 Result of linear mixed-effects model for comport ranking for all of 5 time points of comfort assessment and both electrode types. 9 conditions are compared to the reference condition Multipin sitting after initial cap application.

**Fixed effects – conditions**	**Estimate**	**SE**	**t statistics**	**DF**	**p-value**	**CI lower**	**CI upper**
Intercept	7.3499	0.3688	19.9268	190	0	6.6224	8.0775
Flower – sitting start	1.3000	0.3859	3.3683	190	$0.92*10^{-3}$	0.5387	2.0612
Multipin – sitting end	-2.4999	0.3859	-6.4775	190	$0.78*10^{-9}$	-3.2612	-1.7387
Flower – sitting end	0.8000	0.3859	2.0728	190	$0.39*10^{-1}$	0.0387	1.5612
Multipin – supine start	-2.2999	0.3859	-5.9593	190	$0.12*10^{-7}$	-3.0612	-1.5387
Flower – supine start	-1.2499	0.3859	-3.2387	190	$0.14*10^{-2}$	-2.0112	-0.4887
Multipin – supine end	-4.2999	0.3859	-11.1414	190	0	-5.0612	-3.5387
Flower – supine end	-2.4999	0.3859	-6.4775	190	$0.78*10^{-9}$	-3.2612	-1.7387
Multipin – overall end (sitting)	-2.1499	0.3859	-5.5707	190	$0.86*10^{-7}$	-2.9112	-1.3887
Flower – overall end (sitting)	-0.1999	0.3859	-0.5182	190	0.61	-0.9612	0.5612
**Random effects - individuals**	**Estimate**					**CI lower**	**CI upper**
Intercept	1.1097	-	-	-	-	0.7838	1.5709
Error	1.2204	-	-	-	-	1.1006	1.3532

Supplementary Table S6 Comfort statistics comparison of multichannel EEG studies with dry electrodes. NA – not available.

**References**	**Description**	**Channel number**	**Application duration (min)**	**Acquisition duration (min)**	**Comfort**		
					**Rating**	**Before acquisition**	**After acquisition**
This study	**Flower-shaped**: pin length= 5 mm with hardness A60 (prefrontal and frontal-temporal) and A85; **Multipin** (A98); Pin length: 6 mm, 3mm, and 1.5mm	64	23 ± 5 (Multipin dry), 24 ± 6 (Flower dry)	~ 42 (sit)	10-most comfort, 1-most pain	7.4 ± 1.4 (MD), 8.7 ± 1.1 (FD)	4.9 ± 2.3 (MD); 8.2 ± 1.3 (FD)
				~76 = 42 (sit) + 34 (supine)			5.1 ± 1.3 (MD); 6.1 ± 1.6 (FD)
				~77 (return to sit from supine)			5.2 ± 2.0 (MD); 7.2 ± 1.8 (FD)
Heijs et al. 2021	**Multipin**; Pin length= 1.5 mm (A40, prefrontal), 3 mm (A60, frontal-temporal and anterior), 6 mm (A80)	32	31 ± 10 (gel), 23 ± 13 (dry);	NA	1-most comfort, 10-most pain	2.0±1.0 (gel); 2.0± 1.2 (dry)	2.0±1.3 (gel); 3.0±1.5 (dry)
Ng et al. 2022	**Multipin** (A98); Pin length: 6 mm, 3mm, and 1.5mm	64	32.3 ± 13.8 (gel), 12.4 ± 6.5 (dry);	21.3 ± 9.3 (gel,sit), 23.4 ± 8.3 (dry,sit);	1-most comfort, 10-most pain	2.4±1.3 (gel); 3.6± 1.8 (dry)	2.5±1.4 (gel); 4.3± 2.2 (dry)
Di Fronso et al. 2019	**Multipin**; Pin length: 6 mm, 3mm, and 1.5mm	64	39 ± 18 (gel), 13 ± 3 (dry)	80 (incl. application time,sit and cycling)	1-most comfort, 10-most pain	3 ± 2 (gel); 4 ± 2 (dry);	2 ± 2 (gel); 3 ± 2 (dry);
Fiedler et al. 2022	**Multipin**; Pin length: 6 mm, 3mm, and 1.5mm	256	91 (gel), 47 (dry)	~ 30 (sit)	1-most comfort, 10-most pain	2.5 ± 1.0 (gel); 3.6 ± 1.5 (dry)	3.0 ± 1.1 (gel); 4.0 ± 1.8 (dry)
Vasconcelos et al. 2021	**Arch-shaped** (A93); Arch-width 1mm, outer radius 6mm.	21	22 ± 4 (gel), 15 ± 3 (Arch dry), 5 ± 1 (Multipin dry)	60 (sit)	1-most comfort, 10 - most pain	2.6 ± 0.9 (gel); 2.8 ± 0.8 (arch); 3.1 ± 0.9 (Multipin)	2.7 ± 0.8 (gel); ~3.3 (arch); 4.5 ± 1.1 (multipin)
Hinrichs et al. 2020	**Multipin** with spring loaded pin	19	6.36 ± 1.18 (gel); 4.0 ± 0.7 (dry)	20 (sit)	7-did not notice it, 1-unbearable,	5.64 ± 1.37 (gel); 5.83 ±1.34 (dry)	5.42 ± 1.13 (gel); 5.75 ± 1.08 (dry)
				30 (sit)			4.54 ± 0.73 (gel); 5.69 ± 1.02 (dry)
				60 (sit)			3.24 ± 0.35 (gel); 5.07 ± 0.63 (dry)
Hairston et al. 2014	**Multipin** with spring loaded pin	9	NA	60 (sit)	1-very comfortable and 7-very uncomfortable	NA	2.0 (gel sponge); 5.0 (dry)
